# A case of breakthrough *Candida parapsilosis* fungemia during micafungin therapy for a *Candida glabrata* bloodstream infection

**DOI:** 10.1016/j.mmcr.2017.03.002

**Published:** 2017-03-23

**Authors:** Yuta Norimatsu, Daiichi Morii, Asako Kogure, Taeko Hamanaka, Yoshihiro Kuwano, Takayuki Yokozawa, Toshimi Oda

**Affiliations:** a Department of Infectious Diseases, Showa General Hospital, 8-1-1 Hanakoganei, Kodaira, Tokyo 187-8510, Japan; b Department of Infection Control and Prevention Graduate School of Medicine, Osaka University, 2-15 Yamadaoka, Suita, Osaka 565-0871, Japan; c Department of Dermatology, Showa General Hospital, 8-1-1 Hanakoganei, Kodaira, Tokyo 187-8510, Japan; d Department of Clinical Laboratory, Showa General Hospital, 8-1-1 Hanakoganei, Kodaira, Tokyo 187-8510, Japan

**Keywords:** Breakthrough infection, *Candida parapsilosis*, *Candida glabrata*, Pyoderma gangrenosum, Micafungin

## Abstract

We describe a case of breakthrough Candida parapsilosis fungemia in an 80-year-old woman with pyoderma gangrenosum and rheumatoid arthritis. C. parapsilosis was detected in blood culture while the patient was treated with micafungin for a Candida glabrata bloodstream infection. The breakthrough infection was successfully treated with liposomal amphotericin B.

## 1 Introduction

*Candida* spp. are the fourth leading cause of catheter-related bloodstream infections (BSIs) [1]. Among all *Candida* infections, >90% are caused by *Candida albicans*, *Candida glabrata*, *Candida tropicalis*, *Candida parapsilosis*, and *Candida krusei* [2], [3], [4]. The minimum inhibitory concentrations (MICs) of the echinocandins to *C. parapsilosis* are higher compared with those of most other *Candida* spp., raising the concern that echinocandins might not be the best treatment option for preventing *C. parapsilosis* infections [5], [6]. However, it has also been reported that echinocandins and other antifungals are equally effective in the treatment of *C. parapsilosis* BSIs [7]. We herein report an 80-year-old woman with pyoderma gangrenosum and rheumatoid arthritis in whom a breakthrough infection of *C. parapsilosis* was detected in blood culture during micafungin therapy for a *C. glabrata* bloodstream infection. The breakthrough infection was successfully treated with liposomal amphotericin B.

## 2 Case

An 80-year-old Japanese woman presented to the Dermatology Department of the Showa General Hospital on day 0 with a 1-month history of spreading ulcers due to pyoderma gangrenosum. Her past medical history included pyoderma gangrenosum, diabetes mellitus, and rheumatoid arthritis. Her daily medications included alfacalcidol, salazosulfapyridine, polaprezinc, methotrexate, lansoprazole, folic acid, diclofenac, and felbinac.

On initial physical examination, she had a temperature of 37.0 °C (98.6 °F) and a blood pressure of 111/45 mmHg, with a heart rate of 51 beats per minute. Her height was 145.5 cm, and her body weight was 45.5 kg. The patient presented with several ulcers on all her extremities but had otherwise unremarkable findings. The initial laboratory workup (day 0) showed hyperglycemia of 237 mg/dL, leukocytosis (white blood cell count [WBC], 16,040/µL), and abnormal renal function (blood urea nitrogen [BUN], 39.6 mg/dL; creatinine 1.64 mg/dL). An echocardiogram showed a heart rate of 38 beats per minute and a complete atrioventricular block.

**Fig. 1:**
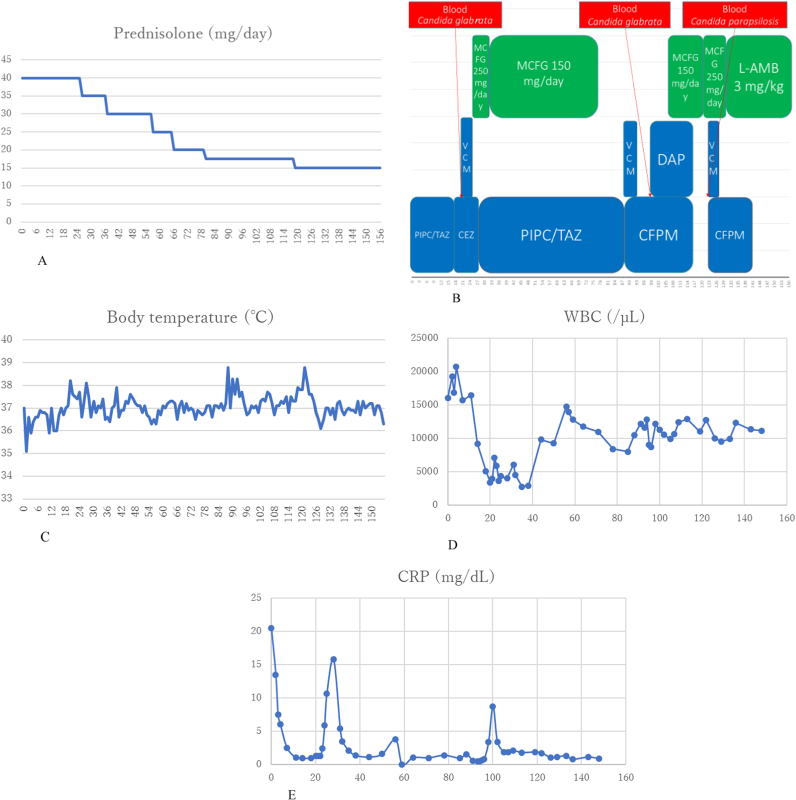
**Clinical course,** A) Prednisolone dosages, B) Medications and dosages, C) Body temperature across time, D) White blood cell counts, E) C-reactive protein levels. CEZ, cefazolin; CFPM, cefepime; CRP, C-reactive protein; DAP, daptomycin; L-AMB, liposomal amphotericin B; MCFG, micafungin; PIPC, piperacillin; TAZ, tazobactam; VCM, vancomycin; WBC, white blood cell count.

The clinical course is shown in Fig. 1. The patient was treated with prednisolone 40 mg/day for pyoderma gangrenosum; moreover, tazobactam/piperacillin was administered since an infection of the ulcers was suspected. On day +19, a pacemaker was implanted to treat the complete atrioventricular block. On day +23, Gram staining of the blood culture showed numerous yeast cells that were identified as *C. glabrata* (Table 1A)*,* on API® ID32C (API-bioMérieux France). Micafungin 250 mg/day was administered (starting on day +23) followed by 150 mg/day (starting on day +29) after exclusion of fungal endophthalmitis. The pacemaker was removed to remove a possible source of infection on day +23. On day +30, a central venous (CV) catheter was inserted; it was subsequently removed and reinserted on day +38. On days +40 and +54, skin grafting was performed for the ulcers. As guidelines state that antifungal treatment should be completed >2 weeks after a negative blood culture (on day +31), micafungin therapy was discontinued on day +77 after a total of 54 days of treatment. On day +88, the CV catheter was removed and then reinserted. On day +105, Gram staining of the blood culture showed the recurrence of numerous yeast cells that were again identified as *C. glabrata* (Table 1B). As this was a recurrence of the *C. glabrata* BSI, we re-administered micafungin 150 mg/day and removed and reinserted the CV catheter. On day+109, another skin grafting procedure was performed. On day +120, Gram staining of the blood culture showed numerous yeast cells yet again; consequently, the micafungin dose was increased to 250 mg/day and the CV catheter was removed and then reinserted. On day +127, the blood culture and the CV catheter culture tested positive for *C. parapsilosis* (Table 1C)*,* identified by API® ID32C (API-bioMérieux France).

**Table 1A:** The MIC of *Candida glabrata*.

**Medicine**	**MIC (mg/L)**
**AMPH-B**	**0.25**
**5-FC**	**<=0.12**
**MCZ**	**0.06**
**FLCZ**	**8**
**ITCZ**	**0.12**
**MCFG**	**<=0.015**
**VRCZ**	**0.25**

**Table 1B:** The MIC of *Candida glabrata*.

**Medicine**	**MIC (mg/L)**
**AMPH-B**	**0.5**
**5-FC**	**<=0.12**
**MCZ**	**0.06**
**FLCZ**	**8**
**ITCZ**	**0.25**
**MCFG**	**0.03**
**VRCZ**	**0.25**

**Table 1C:** The MIC of *Candida parapsilosis*.

**Medicine**	**MIC (mg/L)**
**AMPH-B**	**0.5**
**5-FC**	**<=0.12**
**MCZ**	**0.12**
**FLCZ**	**0.25**
**ITCZ**	**0.06**
**MCFG**	**0.12**
**VRCZ**	**<=0.015**

Micafungin was switched to liposomal amphotericin B (3 mg/kg/day), as this medication is effective for both *C. glabrata* and *C. parapsilosis*. On day+155, liposomal amphotericin B therapy was discontinued after a total treatment duration of 28 days, as the treatment had been provided >2 weeks after the negative blood culture result (day+134). On day+156, the CV catheter was removed and the patient was discharged from the hospital after successful treatment.

## 3 Discussion

We encountered a case of breakthrough *C. parapsilosis* fungemia in a patient who was treated with micafungin for a *C. glabrata* BSI.

Pfeiffer et al. showed that 50% of breakthrough invasive candidiasis cases among patients treated with micafungin were associated with *C. parapsilosis* [8]. However, no other *Candida* spp. had been identified in the patients in their study. To the best of our knowledge, the case presented herein is unique because *C. parapsilosis* was identified in the patient while she was treated for *C. glabrata* fungemia.

The MICs of the echinocandins to *C. parapsilosis* are higher compared with those of most other *Candida* spp. [9]. However, Ruiz et al. reported that echinocandins did not impair the treatment success in patients with *C. parapsilosis* BSIs [7].

Generally, echinocandins can be used to treat *C. parapsilosis* BSIs if the MICs of the echinocandins are below the reference values of the Clinical and Laboratory Standards Institute (CLSI) and the European Committee on Antimicrobial Susceptibility Testing (EUCAST) methods [5]. In this case, the MIC of micafungin was low for *C. parapsilosis* (0.12 mg/L). According to CLSI® M27 S3 [10] and CLSI® M27 S4 [11], a MIC of 0.12 mg/L means that *C. parapsilosis* is susceptible to micafungin. Our case suggests that micafungin should not be the first-line treatment for *C. parapsilosis* BSIs even if the MIC is low enough to be classified as “susceptible”.

*C. parapsilosis* forms biofilms and is often associated with catheter-related BSIs [9], [12], [13], [14]. In our case, *C. parapsilosis* might have been introduced into the patient's body by the CV catheters. Removal of CV catheters is one of the most important therapies to treat candidiasis [11]. We removed the CV catheters every time when Gram staining of the patient's blood culture showed numerous yeast cells. The patient also had pyoderma gangrenosum; thus, ulcers might have also been primary entry sites for *C. parapsilosis*. Thaler et al. reported that wounds can be sources of candidiasis in patients with cancers [15]. Our patient was undergoing prednisolone therapy, and her past medical history included diabetes mellitus. Thus, the patient was considered an immunocompromised host.

In conclusion, we report a case of breakthrough candidemia of *C. parapsilosis.* This suggests that when *C. parapsilosis* is detected in a blood culture, echinocandins might not be the first-line treatment even if the MIC of the echinocandin is below the reference value.

## Conflict of interest

None.

## Ethical form

This study received no funding, and there are no potential conflicts of interest to declare. We obtained written and signed consent to publish the case report from the patient.
